# Moderate mental illness as a predictor of chronic disease prevention and screening

**DOI:** 10.1186/s12875-017-0645-x

**Published:** 2017-06-17

**Authors:** Ginetta Salvalaggio, Christopher Meaney, Rahim Moineddin, Eva Grunfeld, Donna Manca

**Affiliations:** Ginetta Salvalaggio, University of Alberta, Edmonton, Canada

**Keywords:** Primary care, Family practice, Chronic disease prevention, Screening, Depression, Anxiety

## Abstract

**Background:**

Primary care plays a pivotal role in the provision of both mental health care and chronic disease prevention and screening (CDPS). Unfortunately, moderate mental illness (MMI) is associated with poorer general health outcomes. Part of this burden of illness may be due to reduced CDPS uptake. The Building on the Existing Tools to Improve Chronic Disease Prevention in Primary Care (BETTER) trial recruited 777 participants ages 40–65 from 32 family practice panels, of whom 135 (18.2%) had elevated GAD scores, 118 (16.4%) had elevated PHQ scores, and 264 (34.0%) had electronic medical record (EMR)-documented MMI. We hypothesized that patients with screen-positive or chart-documented MMI are 1) eligible for more CDPS actions, and 2) able to complete a lower proportion of CDPS actions than patients unaffected by MMI.

**Methods:**

This study was a secondary analysis of data from the BETTER trial. Participants were stratified by both EMR-documented MMI and screen-positive evidence of MMI (using the General Anxiety Disorders (GAD-7) and Patient Health Questionnaire (PHQ-9) instruments for anxiety and depression screening, respectively). The primary outcome was the proportion of CDPS actions for which the patient was eligible completed at follow-up, using a composite index.

**Results:**

After adjusting for age, gender, and social support, patients with evidence of MMI had a lower composite index than patients without evidence of MMI (*p* < 0.05). The lower composite index is primarily due to higher eligibility for CDPS at baseline; ability to complete CDPS was not statistically different.

**Conclusions:**

Patients affected by MMI are eligible for more CDPS actions than their unaffected counterparts. Although they are able to complete a similar number of CDPS actions, they are not able to eliminate their baseline CDPS gap. Primary care teams need to be aware of this increased CDPS eligibility for patients with MMI and ensure best practices in CDPS supports are available to this patient population. Further study is needed to determine the ideal suite of targeted supports.

## Background

Primary care plays a significant role in the provision of mental health care [1]. Often, family physicians are the initial point of contact for moderate mental illness (MMI) such as anxiety and depression [2]. In addition, treatment of these mental health concerns is usually provided without the assistance of a specialist provider [3–5]. Primary care physicians are also responsible for the management of chronic nonpsychiatric illness and preventive health; it can be difficult to address all chronic medical issues and preventive health actions in traditional family practice visits [6–9]. MMI is commonly associated with higher social complexity, including reduced health literacy, lack of child care or transportation, low income, and unstable or unsafe living environments [10–12]. These factors can impede patients’ ability to complete labwork or eat and exercise according to preventive care guidelines [11–13]. Often, the most pressing issues during any visit to a primary care team are managed, and other issues may be delayed until a subsequent appointment. Issues delayed to another appointment may never be adequately addressed and chronic disease prevention and screening (CDPS) can easily be lost among these other priorities [8, 9, 14].

Literature suggests that adherence to guideline based CDPS actions is not equal among different populations. Patients living with low socioeconomic position, addiction, and/or mental illness are disadvantaged with respect to CDPS, completing a lower number of screening actions compared to the general population [15, 16]. It could also be hypothesized that poorer health outcomes in these groups may be accounted for in part by reduced CDPS. Clinical practice guidelines for specific diseases acknowledge the association between mental and physical illness and encourage clinicians to screen for MMI in the presence of other chronic conditions [17]; however they do not address the converse (screening for chronic conditions in the presence of MMI). Few tools exist to support CDPS uptake for patients with MMI, and no rigorously studied interventions to improve CDPS uptake in this population have been published.

MMI is not uncommon in the primary care setting; a recent report from the World Health Organization found the prevalence of depression in primary care in various countries ranged from 5–20% and anxiety ranged from 4–15% [1]. In Canada, one study estimated that 1 in 5 Canadians are affected annually by MMI [18] and another identified that 14% of primary care patients had a diagnosis of depression [19]. Given the prevalence of MMI in primary care populations, it is particularly important to explore potential differences in CDPS uptake in these patients compared to those without MMI. The purpose of this study was to explore the relationship between eligibility for - and completion of CDPS - actions and MMI. Specifically, we investigated whether those patients with screen-positive or chart-documented MMI are eligible for more not-yet-completed CDPS actions compared to individuals without MMI. Further, we investigated whether those with MMI are able to accomplish CDPS actions for which they are eligible at a similar rate as their unaffected counterparts.

## Methods

### Design, setting and patients

To investigate differences in CDPS between patients with versus without MMI, this study used a subset of data from the Building on the Existing Tools to Improve Chronic Disease Prevention in Primary Care (BETTER) trial [20]. The BETTER trial was a pragmatic factorial randomized controlled trial conducted in eight primary care team practices in two Canadian cities (Toronto, Ontario and Edmonton, Alberta). Within each of the participating primary care teams were four primary care physicians. In total, 32 primary care physicians (and their patients) participated in the BETTER trial (16 from Toronto and 16 from Edmonton). Patients in the BETTER trial were limited to actively rostered patients between the ages of 40–65 (as most CDPS actions target individuals in this age range). The BETTER trial recruited patients based on information derived from the patients’ electronic medical record. Patients were stratified into one of two groups: 1) without MMI and 2) with MMI. MMI was defined by attendance for a MMI diagnosis in the previous 12 months and documentation of 1) depression, anxiety, or pyschosomatic illness in two different places in the chart, 2) two different MMI diagnoses in the same charting field, or 3) one MMI diagnosis and one prescription for psychiatric medication. Forty-five randomly selected patients—25 without chart-documented MMI, and 20 with chart-documented MMI—from each physician practice were invited to participate. Patients documented as unstable or with more severe mental illnesses such as schizophrenia and bipolar affective disorder were excluded from the study.

The survey that patients completed at baseline included the Generalized Anxiety Disorder 7-item scale (GAD-7) and the Patient Health Questionnaire (PHQ-9). Moderate anxiety is more likely with a GAD score greater or equal to 10 [21, 22]; moderate depression is more likely with a PHQ score greater or equal to 10 [23, 24]. Our research team considered the likelihood that a) some patients *with* chart-documented MMI (“detected”) may screen *negative* on both the GAD and PHQ (“inactive symptoms”) as a result of disease remission, and b) some patients *without* chart-documented MMI (“not detected”) may screen *positive* on either the GAD or PHQ (“active symptoms”) due to undetected illness or lack of documentation. In theory, patients with clinically active symptoms would have the highest burden of illness, hence those with both chart-documented and screen-positive MMI would be the most affected by their condition and least able to carry out recommended CDPS actions, followed by patients with chart-documented MMI only, with screen-positive but undiagnosed patients least affected of these three groups and most able to achieve CDPS actions. Thus, for the purposes of this analysis, we stratified BETTER trial participants into four groups: 1) detected / active symptoms; 2) detected / inactive symptoms; 3) not detected / active symptoms; and 4) not detected / inactive symptoms (“unaffected”).

### Outcome measures

The primary outcome of the BETTER trial was a composite endpoint of the proportion of eligible CDPS actions for which patients were eligible at baseline (denominator), achieved at follow-up (numerator). This outcome, a composite index, was modeled after the Summary QUality InDex (SQUID) introduced by Nietert [25]. The 28 items in the composite related to primary prevention and screening for diabetes, cardiovascular disease and cancer, as well as the promotion of a healthy lifestyle (diet, exercise, weight management, smoking, alcohol), supported by clinical practice guidelines [17]. Precise operational definitions of each of the 28 components of the composite endpoint can be found in the article describing the BETTER trial outcomes [20]. At baseline, each patient was assessed to determine (through use of a patient self-report survey and the patient’s electronic medical record (EMR)) their eligibility for each of the 28 components under consideration. Patients were followed prospectively from baseline for six months, at which time actual completion of each action was reassessed.

### Statistical analysis

Descriptive summaries of the data are presented as means and standard deviations for continuous variables; counts and percentages for categorical variables.

We compared our composite index outcome across discrete groups (i.e. the mental health indicator), using a non-parametric Kruskal-Wallis test; we also used generalized estimating equation (GEE) models to adjust for the design effect of patients being clustered within physicians. We present two variations of the GEE model; the first, a bivariate GEE model using only the categorical grouping variable under consideration (i.e. the MMI indicator); and the second, a multivariate GEE model estimating the impact of the same categorical grouping variable on the composite index after adjusting for age, gender, and social support.

We considered individual components of the composite index related to smoking and alcohol as these behaviours are known to be more prevalent in populations affected by MMI [26–30]. As this analysis is exploratory, we only present counts and proportions corresponding to the number of patients eligible and achieving actions in each of the MMI groups.

All statistical analyses were conducted in SAS version 9.3 (Cary, North Carolina).

### Research ethics and trial registration

The trial was approved by the Ontario Cancer Research Ethics Board (REB), University of Alberta REB, and all relevant REBs in each province and at each primary care team site. The registration number of the original RCT BETTER trial was ISRCTN07170460.

## Results

The BETTER trial enrolled 789 patients. Twelve patients withdrew from the trial prior to completion and 777 patients were included in the analysis. Patients enrolled in the BETTER trial were ages 40–65, predominantly female, Caucasian, and well educated. A more detailed description of the trial participants can be found in Grunfeld et al. [20].

Of the patients with available scores, 18.2% (135/742) had elevated GAD scores, 16.4% (118/720) had elevated PHQ scores, and 34.0% (264/777) had chart-documented MMI, with significant overlap between those patients identified via screening and those identified through the medical record (Table 1). Of the 500 patients with available GAD and/or PHQ scores and without chart-documented MMI, a total of 15.0% (75/500) screened positive for MMI as determined by the GAD and the PHQ, with 12.0% (60/500) screening positive on the GAD and 8.2% (41/500) screening positive on the PHQ.

**Table 1:** Stratification of patients by screen-positive and chart-documented evidence of MMI

	Active Symptoms	Inactive Symptoms	
Detected	13%(99) EMR + / Screen +	21%(161) EMR + / Screen -	34%(260) Detected
Not Detected	10%(75) EMR - / Screen +	56%(425) EMR - / Screen -	66%(500) Not Detected
	23%(174) Active	77%(586) Inactive	100%(760) Total

Table 2 summarizes demographic and behavioural characteristics across each of the screen-defined and chart-documented MMI strata. MMI was associated with female gender, lower levels of employment and income, and fewer social supports. Additionally, MMI was associated with higher levels of smoking, more frequent alcohol consumption, and higher levels of obesity.

**Table 2:** Characteristics of patients by baseline screen-positive (GAD-7/PHQ-9) and chart-documented MMI^a^

	Composite Covariate (GAD, PHQ, BETTER MMI)				
	Not Detected / Inactive Symptoms (Unaffected, *N* = 425)	Detected / Inactive Symptoms (*N* = 161)	Not Detected / Active Symptoms (*N* = 75)	Detected / Active Symptoms (*N* = 99)	*P*-value
Age (years)	53.4 (6.9)	53.9 (6.6)	51.3 (6.6)	51.8 (6.4)	0.006
Female sex	282 (66.4)	128 (79.5)	56 (74.7)	80 (80.8)	0.002
Minority race or ethnic group	49 (11.9)	13 (8.2)	10 (13.9)	13 (13.5)	0.47
≥1 yr. post-secondary education	377 (89.1)	130 (80.8)	65 (87.8)	77 (77.8)	0.006
Full or Part-time Employment	334 (79.0)	124 (77.0)	60 (81.1)	54 (55.7)	<0.0001
Married/common-law	350 (82.6)	116 (72.1)	52 (71.2)	57 (58.2)	<0.0001
Total Household Income					
≥ 100,000 CAD	236 (58.4)	76 (47.8)	32 (43.8)	28 (29.8)	<0.0001
60,000–99,999 CAD	109 (27.0)	48 (30.2)	20 (27.4)	29 (30.9)	
0–59,999 CAD	59 (14.6)	35 (22.0)	21 (28.8)	37 (39.3)	
Current smoker	26 (6.1)	12 (7.5)	8 (10.7)	33 (33.3)	<0.0001
Current alcohol consumption					
< 4× per month	260 (61.3)	105 (65.2)	52 (71.2)	72 (73.4)	0.04
≥ 4× per month and <2× per week	111 (26.2)	29 (18.0)	12 (16.4)	13 (13.3)	
≥ 2× per week	53 (12.5)	27 (16.8)	9 (12.4)	13 (13.3)	
Exercise status					
Extremely active	82 (19.3)	29 (18.2)	7 (9.6)	16 (16.3)	0.24
≤ Mildly active	342 (80.6)	130 (81.8)	66 (90.4)	82 (83.7)	
Body-mass index	24.9 (4.9)	25.3 (5.2)	26.5 (5.5)	26.8 (5.8)	0.004
Obese	58 (14.1)	27 (16.9)	16 (22.2)	29 (29.3)	0.003
MOS Social Support Score	83.4 (18.3)	75.2 (23.3)	60.7 (27.1)	55.4 (28.6)	<0.0001

^**a**^ Continuous variables presented as means (SD) and categorical variables presented as N (%). Kruskal Wallis tests are used for continuous variables, and Person’s chi-squared tests or Fisher’s exact tests are used for categorical variables

CAD: Canadian dollars

MOS Social Support Questionnaire: Scores for the 22 items range from 0 to 100, higher scores indicate higher level of self-perceived support

Patients with the most active MMI accomplished the same number of, but were eligible for more, actions than their less affected counterparts (Table 3). The “detected/active symptoms” group (*N* = 99, composite index = 36.0%, SD = 27.5%) had a statistically lower mean composite index than each of the other three groups (*p* < 0.05). The mean composite indices for “detected/inactive symptoms” (*N* = 161, composite index = 41.7%, SD = 27.9), “not detected/active symptoms” (*N* = 75, composite index = 41.1%, SD = 27.9), and “unaffected” (*N* = 425, composite index = 45.3%, SD = 27.8) groups were not statistically different from one another.

**Table 3:** Estimates of Composite Index by baseline screen-positive (GAD-7/PHQ-9) and chart-documented MMI

	Composite Covariate (GAD, PHQ, BETTER MMI)				
	Not Detected / Inactive Symptoms (Unaffected, *N* = 425)	Detected / Inactive Symptoms (*N* = 161)	Not Detected / Active Symptoms (*N* = 75)	Detected / Active Symptoms (*N* = 99)	*P*-value
Total Composite					
Unadjusted Means	45.3 (42.6, 48.0)	41.7 (37.4, 46.1)	41.1 (34.7, 47.6)	36.0 (30.5, 41.5)	0.02
Bivariate GEE^a^	43.2 (36.5, 49.9)	40.6 (33.9, 47.3)	40.2 (32.3, 48.0)	32.8 (25.7, 39.9)	0.02
Multivariate GEE^b^	43.1 (36.4, 49.9)	41.2 (34.4, 48.0)	41.1 (33.1, 49.1)	34.2 (26.9, 41.5)	0.03
Eligible (E)					
Unadjusted Means	8.8 (8.5, 9.0)	9.1 (8.6, 9.6)	9.1 (8.4, 9.8)	10.3 (9.6, 10.9)	0.0003
Bivariate GEE^a^	8.7 (8.3, 9.1)	9.2 (8.6, 9.7)	9.1 (8.4, 9.9)	10.1 (9.4, 10.9)	0.06
Multivariate GEE^b^	8.7 (8.3, 9.1)	9.1 (8.6, 9.6)	9.2 (8.6, 9.9)	10.2 (9.5, 11.0)	0.048
Met (M)					
Unadjusted Means	3.9 (3.6, 4.2)	3.8 (3.4, 4.3)	3.9 (3.3, 4.6)	3.8 (3.2, 4.4)	0.87
Bivariate GEE^a^	3.7 (3.2, 4.3)	3.8 (3.2, 4.6)	3.9 (3.1, 4.9)	3.4 (2.7, 4.3)	0.45
Multivariate GEE^b^	3.7 (3.2, 4.3)	3.8 (3.2, 4.6)	4.1 (3.3, 5.1)	3.6 (2.9, 4.5)	0.51

^a^adjusting for clustering effect in design and single main effect

^b^adjusting for clustering effect in design as well as age, gender and social support

We additionally examined the six alcohol / tobacco screening, monitoring and referral components of the composite index, in terms of both eligibility for these actions (E, Table 4) and meeting these actions (M, Table 5). The presence of MMI was associated with increased eligibility for most smoking and alcohol monitoring and referral actions. A significant difference was not observed with respect to screening actions; nor were clear differences noted with respect to meeting any actions for which patients were eligible.

**Table 4:** Unadjusted Eligible (E) table for smoking and alcohol actions

	Composite Covariate (GAD, PHQ, BETTER MMI)				
	Not Detected / Inactive Symptoms (Unaffected, *N* = 425)	Detected / Inactive Symptoms (*N* = 161)	Not Detected / Active Symptoms (*N* = 75)	Detected / Active Symptoms (*N* = 99)	*P*-value
Smoking – Screening	92 (21.7)	33 (20.5)	18 (24.0)	18 (18.2)	0.76
Smoking – Monitoring	38 (8.9)	14 (8.7)	10 (13.3)	35 (35.4)	0.02
Smoking – Referral	38 (8.9)	14 (8.7)	10 (13.3)	35 (35.4)	0.02
Alcohol – Screening	129 (30.4)	49 (30.4)	26 (34.7)	23 (23.2)	0.23
Alcohol – Monitoring	75 (19.5)	36 (24.8)	13 (20.0)	26 (32.5)	0.06
Alcohol – Referral	75 (19.5)	36 (24.8)	13 (20.0)	26 (32.5)	0.06

**Table 5:** Unadjusted Met (M) table for smoking and alcohol actions^a^

	Composite Covariate (GAD, PHQ, BETTER MMI)				
	Unaffected	Detected / Inactive Symptoms	Not Detected / Active Symptoms	Detected / Active Symptoms	*P*-value
Smoking – Screening	60/92 (65.2)	21/33 (63.6)	12/18 (66.7)	12/18 (66.7)	0.61
Smoking – Monitoring	22/38 (57.9)	3/14 (21.4)	4/10 (40.0)	9/35 (25.7)	0.046
Smoking – Referral	8/38 (21.1)	4/14 (28.6)	5/10 (50.0)	11/35 (31.4)	0.21
Alcohol – Screening	72/129 (55.8)	29/49 (59.2)	9/26 (34.6)	12/23 (52.2)	0.23
Alcohol – Monitoring	44/75 (59.5)	23/36 (74.2)	7/13 (58.3)	17/26 (70.8)	0.27
Alcohol – Referral	8/75 (10.7)	5/36 (13.9)	4/13 (30.8)	6/26 (23.1)	0.33

^**a**^Where denominator for each cell = those participants eligible (E) for each action (see Table 4)

## Discussion

The purpose of this study was to assess potential differences in eligibility for, and adherence to, guideline based CDPS actions in patients with and without MMI. Patients with screen-positive and chart-documented MMI are eligible for more CDPS actions than their unaffected counterparts and complete a similar number of actions [20]. This suggests that the nature of MMI places patients at a health promotion disadvantage.

Of the 500 patients with available GAD and/or PHQ scores and without chart-documented MMI, 15% (75/500) scored positive on the PHQ and/or GAD, supporting Craven’s findings that primary care detection rates for MMI may be low [2]. However, screen positive patients without chart-documented MMI (“not detected/active symptoms”) had similar composite indices to unaffected patients. This finding fails to support more universal primary care *screening* for MMI as a means to improve CDPS uptake.

In contrast, patients with chart-documented MMI who screened positive on the PHQ and/or GAD (“detected/active symptoms”) had lower composite indices than unaffected patients, whereas patients with chart-documented MMI who screened negative (“detected/inactive symptoms”) had similar composite indices to unaffected patients. This suggests that *treatment* of clinically significant MMI may have a potential impact on CDPS uptake; however, MMI treatment has yet to be confirmed in a prospective trial setting as an effective intervention for CDPS uptake.

For those patients with the highest burden of illness (detected and with active symptoms of MMI), there is increasing evidence of the cost effectiveness of interventions to promote the physical health of people with MMI [31]. Patients with MMI may particularly benefit from individualized patient-level support from a care team member, perhaps with special emphasis on health navigation and motivational interviewing as demonstrated by the BETTER trial [20]. Although screening asymptomatic patients may not be justified, we propose that primary care teams be vigilant for signs of MMI and maintain awareness of its impact on CDPS.

Our findings that MMI was associated with female gender, lower levels of employment and income, and fewer social supports are consistent with the literature [11]. Also, there is a known increased prevalence of smoking and excessive alcohol intake among patients with MMI, and vice versa [26–30]; smoking and alcohol-related CDPS uptake may be particularly affected by MMI. Teams would do well to address potential socioeconomic barriers to carrying out CDPS actions. Targeted CDPS interventions, designed with MMI-associated CDPS barriers in mind, are needed, but require evaluation of their effectiveness and relative cost prior to large scale implementation.

There exist potential limitations that should be considered when interpreting results from this study. First, as a pragmatic trial the BETTER trial employed minimal inclusion/exclusion criteria, and the demographics of the participating patients are not entirely representative of the general urban Canadian population, thus will have limited generalizability to rural, non-Caucasian, or socioeconomically disadvantaged settings. That said, the characteristics of the MMI-affected patients enrolled in this study are similar to those reported in similar literature.

Secondly, the GAD-7 and PHQ-9 are not sufficient information to confirm the diagnosis of a MMI, and rely on self-report by participants. Use of these proxies without confirmation via clinician assessment may not necessarily be the most accurate reflection of patients who are living with untreated illness.

Lastly, the study was not designed with sufficient power to demonstrate a difference in CDPS uptake on the basis of whether or not patients with evidence of MMI symptoms had come to the attention of a physician. Similarly, although trends for increased need for smoking and alcohol CDPS were seen, we were unable to confirm any clear pattern on the basis of MMI status.

## Conclusions

Compared to patients unaffected by MMI, patients with documented MMI and active symptoms complete a smaller proportion of CDPS actions for which they are eligible, and are eligible for more CDPS actions overall. Primary care teams should be aware of this CDPS gap and remain vigilant for patients with active MMI in need of CDPS support. Further research is required to better understand effective approaches to CDPS in patients with MMI, and if diagnosis and treatment of MMI can improve CDPS in primary care.

## Abbreviations

BETTER: Building on the existing tools to improve chronic disease prevention in family practice
CAD: Canadian dollars
CDPS: Chronic disease prevention and screening
E: Eligible actions
EMR: Electronic medical record
GAD: Generalized anxiety disorder
GEE: Generalized estimating eq.
M: Met actions
MMI: Moderate mental illness
PHQ: Patient health questionnaire
REB: Research ethics board
